# Coronary Anomalies in 11,267 Southwest Chinese Patients Determined by Angiography

**DOI:** 10.1155/2021/6693784

**Published:** 2021-02-18

**Authors:** Xin Jiang, Ping Zhou, Chunlan Wen, Zhao Yin, Tao Liu, Meiling Xu, Chengming Yang, Hongyong Wang, Wenxing Song, Yuqiang Fang, Chunyu Zeng

**Affiliations:** ^1^Department of Cardiology, People's Hospital of Dadukou, Dadukou District, Chongqing 400080, China; ^2^Department of Cardiology, Chongqing Institute of Cardiology, Daping Hospital, Army Medical University, Chongqing 400042, China; ^3^Department of Cardiology, The First People's Hospital of Chongqing Liang Jiang New Area, Chongqing 401121, China; ^4^Department of Cardiology, 306th Hospital of PLA, Beijing 100101, China; ^5^Chongqing Emergency Medical Center, Chongqing University Central Hospital, Chongqing 400014, China; ^6^Department of Cardiology, People's Hospital of Dazhu, Dazhu District, Chongqing 402360, China

## Abstract

**Background:**

The prevalence of coronary artery anomalies (CAAs) is rare and varies among different countries or areas. More importantly, the symptoms exhibited by some CAAs make the diagnosis of coronary artery disease (CAD) difficult and hamper the physician from making the right intervention for CAD patients.

**Objective:**

To investigate the prevalence of CAAs in 11,267 patients from three hospitals in Southwest China.

**Methods:**

11,267 patients who have undergone coronary angiography from three Southwest China hospitals were investigated retrospectively. Dominance patterns, prevalence, and the location of each CAA were recorded and analyzed.

**Results:**

The presence of a dominant right coronary artery (RCA) was found in 60.58% of patients. CAAs were found in 11.12% (1258) patients, and 87.66% anomalies were located in the left anterior descending (LAD) artery and its branches. Most of CAAs were found to be myocardial bridges (MBs, 1060 cases, 9.41%). Other CAAs included anomalous coronary origin (43 cases, 0.38%), coronary artery fistulas (CAFs, 36 cases, 0.32%), and coronary artery aneurysm or ectasia (119 cases, 1.06%). It also noted that most anomalies were found with RCA originating from the left coronary sinus (79.07%), most CAFs were located in the LAD and its branches (58.33%), and most coronary artery ectasias were located in the RCA (43.25%).

**Conclusions:**

CAAs in patients from Southwest China were unique compared to other studies. Recognition of these CAAs is important for accurate diagnosis and treatment choice of patients with chest pain.

## 1. Introduction

Coronary angiography (CAG) [1] has traditionally been utilized to detail the coronary vasculature before intervention and surgery and remains the reference standard of imaging modality. Although it has some inherent disadvantages, the high accuracy and lower risk of CAG have made it a useful tool for the diagnosis of coronary artery disease (CAD). With the benefit of CAG, the number of patients who receive percutaneous coronary intervention (PCI) is increasing fast. In China, for example, the number of PCIs performed in 2011was 341,069 which increased to 454,505 in 2013 [2]. This showed an increase of 113,436 patients in 2 years. Therefore, it is important to distinguish a normal image from that of an anomaly of the coronary artery for accurate reference in physician clinics.

Traditionally, coronary arteries are anatomically categorized into 3 groups based on their anatomical features [3], i.e., normal coronary anatomy, anatomical variations of the coronary artery, and coronary artery anomaly (CAA). The frequency of coronary anomalies varied in different countries or regions. For example, the absence of the left main coronary artery was about 0.4%–8% of the total population in Turkey and Singapore [4, 5]; the frequency of coronary artery anomaly was 2.34% in native Chinese Hans and 3.93% in native Chinese Uighurs [6].

It is known that, besides coronary stenosis, many diseases could lead to chest pain. The anomaly of the coronary artery is a major one, which includes severely compressed myocardial bridges, coronary fistulas, coronary aneurysm, and congenital absence of the main coronary artery. Although the prevalence of these anomalies is rare, CAAs could lead to severe complications, make the diagnosis of CAD difficult, and hamper the ability of the physician to perform the correct intervention for patients with CAD [7, 8]. As a representative city in Southwestern China, Chongqing is famous for its mountainous geography, diverse ethnic groups, and massive immigration from other areas of China. However, the prevalence of CAAs in Southwest China is not known. In this present study, we investigated these characteristics, including coronary dominance pattern and prevalence of CAAs, in patients from 3 hospitals in the southwestern region of China, who had undergone CAG, and compared our results with the reported data from different races or regions.

## 2. Methods

### 2.1. Patients

As we reported previously [9], 11,267 patients (4830 females, 6437 males) from Southwest China participated in this research, which lasted from January 2010 to November 2014. They were patients ranging in age from 22 to 92 years (63.4 ± 13.8 years), who were admitted to the 3 southwestern hospitals to undergo CAG. These patients exhibited chest pains, shortness of breath, palpitations, and arrhythmias and were suspected to have CAD. The baseline clinical characteristics of all these patients are shown in Table 1 [9]. The study protocol conforms to the ethical guidelines of the 1975 Declaration of Helsinki as reflected in an a priori approval by the institution's human research committee. Also, all patients have given their informed consent prior to their inclusion in the study.

### 2.2. Procedure of Selective Coronary Angiography

As shown in the literature [9, 10], standard transradial techniques were used for angiography. Lidocaine was used as the local anesthesia. The right radial artery was cannulated using a 21-gauge needle and a transradial kit with a 30-centimeter- (cm) long, 6-French (F) introducer (Terumo, Tokyo, Japan). With the introducer in place, 200 *μ*g nitroglycerin and 2000 IU heparin were introduced into the side port of the sheath. If the operation lasted for more than 1 hour, another dose of heparin (1000 IU/h) was administered intravenously. A 5 F multipurpose catheter (Terumo, Tokyo, Japan) 130 cm long was alternatively used for selective angiography using standard techniques. Briefly, the catheter tip was placed on the orifice of the target artery (the left or the right coronary artery) and introduced with a 0.035″ hydrophilic guide wire (Terumo, Tokyo, Japan). We used iopromide 370 (Bayer, Bayer Schering Pharma, Germany) to take magnified angiography images at different angles. If the angiography failed when using the 5 F multipurpose catheter, the 6 F Judkins catheters (J6F, Cordis Corporation, USA) or 6 F pig catheters (Cordis Corporation, USA) would be used instead. If the radial artery was too small or too circuitous for catheters to manipulate, the femoral artery would be used instead.

### 2.3. The Classification and Definition for Coronary Dominance and Coronary Artery Anomaly

Coronary dominance is defined as the artery whose branches supply the blood of the posterior ventricular wall. Traditionally, the classification of CAAs was based according to Angelini et al.'s methods [11], namely, (a) anomalous pulmonary origins of the coronary arteries, (b) anomalous aortic origins of the coronary arteries, (c) congenital atresia of coronary arteries, (d) myocardial bridging, (e) CAFs, (f) coronary artery aneurysms, and (g) coronary stenosis.

Myocardial bridge (MB) [12] refers to a small segment of the coronary artery tunneling under the myocardium rather than resting on top of it, which presents stenosis compressed by the myocardium during the systole period and presents a normal beat during the diastole period. In the present study, the compressed stenosis of the MB segment was divided into 3 levels: mild (<50%), middle (50-75%), and severe (≥75%). CAFs refer to the abnormal connection between the coronary artery and other structures, including the coronary vein, heart chamber, or pulmonary artery. According to Altin et al. [4], coronary artery ectasia is an abnormal dilatation of the coronary artery segment with the dilated segment 1.5 to 2 times of the adjacent segment, while coronary aneurysm refers to the dilated segment that is more than 2 times of the adjacent segment.

### 2.4. Statistical Analysis

The angiography images of these patients were analyzed by 2 independent investigators. It is noted that those patients with CAAs that occur as a part of complex congenital heart diseases were excluded from this study.

Data and categorical variables were presented as counts or percentages. Statistical analysis was performed by using the two-tailed paired Student *t*-test and chi-square test. A *P* value < 0.05 was considered significant. Statistical analyses were performed using SPSS version 18.0 (SPSS Inc., USA).

## 3. Results

### 3.1. Distribution of Coronary Dominance

As shown in Table 1 [9], 11,267 (6437 males, 4830 females) patients were subjected to diagnostic coronary angiography with mean age of 63.4 ± 13.8 years. Most patients presented with RCA dominance (60.58%, *P* < 0.001), with about 27.51% patients being of the balanced type and only 11.91% patients were LCA dominant (Table 2).

### 3.2. Profile of Coronary Artery Anomalies

As shown in Table 3, the total prevalence of CAAs was 11.22% (1258 cases); MB took the majority of CAAs in Southwest Chinese patients (1060 cases, 9.41%). According to the modified classification [13], the prevalence of CAAs was 0.7% (79/11267) when MB and coronary artery aneurysms/ectasias were excluded. As to artery location, most CAAs were located in LAD and its branches (1108 cases, 88.07%). According to the modified classification with MBs and coronary artery aneurysms/ectasias excluded, the CAAs in the RCA were the main prevalence (50 cases), and the majority of CAAs were of coronary origin anomaly (43 cases, 0.38%).

### 3.3. The Distribution of Myocardial Bridges

There were 1060 patients (9.41%) with MB segments, each patient having 1 MB, except for 2 patients who had 2 MB segments in LAD. The distribution of MBs is shown in Table 4. Most MBs were located in LAD (1043 segments, 98.21%), the left circumflex artery (LCX) had about 1.23% (13 segments), the branch of LAD had about 0.38% (4 segments), and RCA had about 0.19% (2 segments). It was noted that most MB segments were mildly compressed (787 segments, 74.1%), middle compression was intermediate (187 segments, 17.61%), and severe compression (88 segments, 8.29%) was the least.

^##^ *P* < 0.001 compared with other arteries; ^∗∗^*P* < 0.001 compared with other compressed levels. MBs: myocardial bridges; LMCA: left main coronary artery; LAD: left anterior descending; LCX: left circumflex artery; RCA: right coronary artery.

### 3.4. The Distribution of Coronary Origin Anomaly

Forty-three cases (0.38%) of abnormal coronary origin were found in this study. As shown in Table 5, the most original anomaly is RCA, originating from the left coronary sinus (34 cases, 79.07%), while other original anomalies were RCA originating from the ascending aorta (3 cases, 6.98%) and absent left main coronary artery (LMCA) (2 cases, 4.65%). Only 1 case was found with the right conus branch originating directly from the right coronary sinus with an absent LCX. The LMCA originated from the right coronary sinus, with the LCX originating directly from the left coronary sinus.

### 3.5. The Distribution of Coronary Artery Fistulas

There were 36 patients (0.32%) with CAFs. As shown in Table 6, CAFs mainly occurred in 4 coronary arteries: LAD (12 cases, 33.33%), LCX (3 cases, 8.33%), RCA (12 cases, 33.33%), and the diagonal branch of LAD (9 cases, 25%). LAD and its branches were the major artery found with CAFs (21cases, 58.33%). The CAFs entered on the left atrium (12 cases, 33.33%), right ventricle (7 cases, 19.44%), cardiac veins (7 cases, 19.44%), right atrium (4 cases, 11.11%), pulmonary artery (3 cases, 8.33%), left ventricle (2 cases, 5.55%), or aorta (1 case, 0.28%).

### 3.6. The Distribution of Coronary Artery Aneurysms or Ectasias

There were 113 patients (1.00%) suffering from coronary artery ectasias, and only 6 patients (0.05%) were presented with coronary aneurysms. The coronary aneurysms were found in the LAD (4 cases) and the LCX (2 cases). As shown in Table 7, the coronary artery ectasias (CAEs) mainly affected 4 coronary arteries: RCA (50 cases, 43.25%), LAD (34cases, 33.33%), LCX (25 cases, 22.12%), and the diagonal branch of LAD (4 cases, 3.54%).

## 4. Discussion

In the present study, we investigated the CAAs in patients from Southwest China and found that the prevalence was 11.12%. According to the modified classification [13], after exclusion of MBs and coronary artery aneurysms/ectasias, the prevalence was reduced to 0.7% (79/11267). Among CAAs, the myocardial bridge was the most common, which is consistent with Cademartiri et al.'s report [14]. It is noted that our findings are not completely consistent with other reports (As shown in Table 8). For example, 60.58% of patients in our study had RCA dominance, while other reported studies indicated the prevalence at more than 80% [14]. After exclusion of MB and coronary ectasias according to the modified classification [13], the prevalence of CAAs was only 0.7%, which is lower than what was reported in Pan et al.'s study [6], which also showed that CAA frequency was 2.34% for the Chinese Han and 3.93% for the Chinese Uyghur people [6]. The reported prevalence of CAAs varied from 0.4% to 8.47% in different countries or areas [11, 14–19]. Moreover, the abnormal origination of the coronary artery varies among different populations. In our study and those from India, the most abnormal origin is from the RCA [19, 20], while in Turkey and the Netherlands, the most common is from the left main artery (LMA) [11, 14–16], from the LCX for Turkey [17], and from the LAD for Hungary [18].

The myocardial bridge was the major anomaly in our present study as well as in others [14]. We found that most MB segments are located in the LAD, with only a few located in the LCX, branch of the LAD and RCA. These results were similar to the other studies (0.83~11.9%) using coronary angiography [14, 21–24]. In Beijing, Ma et al. found that the prevalence of MBs was 13.6% (336/2462) from the China-Japan Friendship Hospital [25]. In Northeast China, it was reported to be 10.53% (10/95) from Heilongjiang Province [26] and 24.14% (140/580) from Liaoning Province [27]. Our study showed that the frequency of Southwest China was lower as compared with Heilongjiang Province and Liaoning Province in Northeast China. It is known that there are different methods used to diagnose the presence of a myocardial bridge. Other than coronary angiography, coronary computed tomographic angiography is widely used due to its noninvasive characteristics. Of course, autopsy of the heart is the ultimate method for the diagnosis of CAAs. However, the prevalence of CAAs using these methods shows a big difference. For example, coronary computed tomographic angiography shows the frequency of MBs at 22.5% (39/350) in patients from Saudi Arabia [22], 10.86% (39/350) from the Netherlands [14], 44% (108/245) from New York [23], and 26% (39/350) from Israel [24]. Heart autopsy shows the frequency at 34.5% (69/200) in patients from Warsaw [28]. Although the reasons leading to the difference are complicated, the sensitivity of the method used and the different populations are the major ones. Due to its noninvasive nature, more and more people would prefer coronary CT angiography (CTA) examination, even in some patients without any obvious symptoms. In some areas, CTA is taken as a routine procedure during physical examination, although abuse of CTA has escalated in the past few years.

The abnormal origin of the coronary artery is another commonly observed CAA. Similar to the myocardial bridge, the results are different among countries or areas. In China and India [19, 20], the major abnormal origin of the coronary artery is the RCA, whose prevalence varies from 0.38% to 1.3%. In Europe and other countries, the major abnormal coronary artery origin is the left coronary artery (including the left main artery [11, 14–16], LCX [17], and LAD [18]) with a frequency of 0.4~3.3%. Abnormal origin of the coronary artery might elicit some symptoms, with the anomaly of the LMA or LAD showing more clinical symptoms than that of the LCX [22]. Although other abnormal origins of the coronary artery are rare, the consequences are serious, even leading to death during infancy [29–31]. For example, coronary artery originating from the pulmonary artery leads to almost 90% of patients dying during infancy. LMA is the most common, with the LAD and RCA being less common. For those who survive, this anomaly may eventually cause angina, myocardial infarction, and heart failure because of the retrograde blood flow and a left to right shunt, thus requiring the immediate need to be repaired by surgery [29]. In our present study, we did not find any severe abnormal origin of the coronary artery, which might be ascribed to the fact that most patients in our study were adults.

CAF is a fault in the connection between the coronary artery and another vessel or chamber, and the frequency of CAFs is 1/50,000 at birth and 1/500 after cardiac catheterization [13, 17]. Previous studies reported that more than half of the CAFs involved the RCA [13, 17]. However, in the present study, we found the most CAFs involved the LAD and its branches. Moreover, inconsistent with previous reports showing that most of CAFs shunt into the right ventricle and right atrium [15], our present study showed a scattering of the CAF shunt into the left atrium (13 cases, 33.33%), right ventricle (7 cases, 19.44%), cardiac veins (7 cases, 19.44%), right atrium (4 cases, 11.11%), pulmonary artery (3 cases, 8.33%), left ventricle (2 cases, 5.55%), and aorta (1 case, 0.28%). Although the CAF in our study is small and asymptomatic, some reported CAFs are large and may result in pulmonary hypertension, congestive heart failure, bacterial endocarditis, rupture, or myocardial ischemia [17].

Coronary artery ectasia and aneurysm are another rare form of anomaly. They may be associated with inflammatory, connective tissue and some other congenital diseases [11, 32–34]. They were usually considered to be variants of coronary atherosclerosis [15]. In the present study, the frequency of coronary ectasia was 1.00% and coronary aneurysm was 0.05%, which are lower than those found in other studies [11, 32]. Because of the similar prognosis as with atherosclerosis, it was recommended to treat these patients as those with CAD [11, 32–34].

## 5. Conclusion

This study is the first to investigate a relatively large scale of patients in Southwest China and thus shows valuable information. The prevalence of the four CAAs is as follows: MB (9.41, 1060 cases), origin anomaly (0.38, 43 cases), CAFs (0.32, 36 cases), and aneurysms or ectasias (1.06, 119 cases), and most CAA anomalies were located in the LAD artery and its branches. The limitation of this work is that only three hospitals in Southwest China were involved; more hospitals with a larger scale of patients are needed to affirm the finding of this investigation in the future.

## Figures and Tables

**Table 1:** Demographic characteristics and clinical features among patients.

Clinic characteristics	No. of patients (%)
Male/female	6437/4830
Age(years)	22-92 (63.4 ± 13.8)
Clinical presentations	
Typical angina	6138 (54.47)
Atypical chest pain^∗^	2094 (18.58)
Acute coronary syndrome	819 (7.27)
Syncope	372 (3.31)
Arrhythmia or palpitation	725 (6.43)
Recheck the stent	1523 (13.52)
Risk factors for CAD	
Hypertension	4893 (43.43)
Diabetes mellitus	4027 (35.74)
Smoking	3628 (32.2)
Hyperlipidemia	2937 (26.07)
Family history of CAD	4288 (38.06)

^∗^Atypical chest pain: chest pain not caused by myocardial ischemia. CAD: coronary artery disease.

**Table 2:** Distribution of coronary dominance.

Coronary dominance	No. of patients (%)
RCA dominance	6825 (60.58)
Balanced type	3100 (27.51)
LCA dominance	1342 (11.91)

**Table 3:** Profile of CAAs among 11267 patients.

Coronary artery anomaly	LMCA	LAD and branches	LCX and branches	RCA and branches	Total case no. (no/11267∗100%)
MBs		1045	13	2	1060 (9.41)^∗∗^
Origin anomaly	3		2	38	43 (0.38)
CAFs		21	3	12	36 (0.32)
Aneurysms or ectasias		42	27	50	119 (1.06)
Total case no (no/1258∗100%)	3 (0.24)	1108 (88.07)^##^	45 (3.58)	102 (8.11)	1258

^∗∗^ *P* < 0.001 compared with other CAA groups; ^##^*P* < 0.001 compared with other arteries. Total prevalence of CAAs was 11.22% (1258 cases) and MB took the majority (1060 cases, 9.41%). According to the modified classification [13], the prevalence of CAAs was 0.7% (79/11267) when myocardial bridges (MBs) and coronary artery aneurysms/ectasias were excluded. CAAs: coronary artery anomalies; MBs: myocardial bridges; CAFs: coronary artery fistulas; LMCA: left main coronary artery; LAD: left anterior descending; LCA: left coronary artery; LCX: left circumflex artery; RCA: right coronary artery.

**Table 4:** Distribution of myocardial bridges.

MB segment	LAD	LCX	RCA	Branch of LAD	Total no. (%)
Mild compressed (<50%)	772	13	1	1	787 (74.1)^∗∗^
Middle compressed (50~75%)	185	0	1	1	187 (17.61)
Severely compressed (≥75%)	86	0	0	2	88 (8.29)
Total	1043 (98.21)^##^	13 (1.23)	2 (0.19)	4 (0.38)	1062

**Table 5:** Details of coronary origin anomaly.

	No. of cases	Prevalence of origin anomaly (%)
RCA originated from left coronary sinus	34	79.07^∗^
RCA originated from ascending aorta	3	6.98
Right conus branch originated directly from right coronary sinus	1	2.32
LMCA absence	2	4.65
LMCA originated from right coronary sinus	1	2.32
LCX absence	1	2.32
LCX originated directly from left coronary sinus	1	2.32
Total	43	100

^∗^ *P* < 0.01 vs. other abnormal origins. RCA: right coronary artery; LMCA: left main coronary artery; LCX: left circumflex artery.

**Table 6:** Distribution of coronary artery fistulas.

Fistulas	LAD-	LCX-	RCA-	Diagonal of LAD-	Total
Left atrium	8 (22.22)			4 (11.11)	12 (33.33)
Left ventricle				2 (5.55)	2 (5.55)
Right atrium			4 (11.11)		4 (11.11)
Right ventricle	1 (0.28)		6 (16.67)		7 (19.44)
Cardiac vein	1 (0.28)	2 (5.55)	2 (5.55)	2 (5.55)	7 (19.44)
Pulmonary artery	2 (5.55)	1 (0.28)			3 (8.33)
Aorta				1 (0.28)	1 (0.28)
Total	12 (33.33)	3 (8.33)	12 (33.33)	9 (25)	36 (100)

LAD: left anterior descending; LCX: left circumflex artery; RCA: right coronary artery.

**Table 7:** Distribution of coronary artery ectasias.

	No. of patients	Prevalence of coronary ectasias (%)
RCA	50	44.25
LAD	34	30.09
LCX	25	22.12
Diagonal branch of LAD	4	3.54
Total	113	100

**Table 8:** Prevalence distribution of coronary major artery anomalies in different countries.

Literature	Country	Sample size	Major artery (percentage)	Prevalence of CAAs except MB and ectasias, no. (%)	Major CAA (no. %, artery)
Present study	Southwest China	11,267	RCA (60.58)	79 (0.7)	MB (1,060, 9.41, LAD) Origin (43, 0.38, RCA)^∗^
Pan et al. [6]	Xinjiang, Chinese Han, Chinese Uyghur	4746 1934		111 (2.34) 76 (3.93)	Origin (23, 0.48, RCA) Origin (19, 0.98, RCA)
Altin et al. [4]	Turkey	5548	RCA (81.6)	78 (1.4)	Origin (68, 1.2, LMA)
Cademartiri et al. [14]	Netherlands	543	RCA (86.6)	46 (8.47)	MB (59, 10.86, LAD) Origin (18, 3.3, LMA)^∗^
Aydinlar et al. [15]	West Turkey	12,059		100 (0.8)	Origin (48, 0.40, LMA)
Safak et al. [16]	Izmir, Turkey	16,768		120 (0.7)	Origin (86, 0.51, LMA)
Göl et al. [17]	Turkey	58,023		257 (0.4)	Origin (203, 0.35, LCX)
Kardos et al. [18]	Hungary	7694		103 (1.3)	Origin (98, 1.27, LAD)
Garg et al. [19]	India	4100		39 (1.0)	Origin (35, 0.85, RCA)
Zheng et al. [20]	Nanjing, China	1879			Origin (24, 1.3, RCA)

^∗^According to the modified classification [13], myocardial bridge and coronary ectasias were excluded.

## Data Availability

The data used to support the findings of this study are included within the article.
